# Analysis of knowledge of the general population and health professionals on organ donation after cardiac death

**DOI:** 10.5935/0103-507X.20160043

**Published:** 2016

**Authors:** Ramon Correa Bedenko, Renato Nisihara, Douglas Shun Yokoi, Vinícius de Mello Candido, Ismael Galina, Rafael Massayuki Moriguchi, Nico Ceulemans, Paolo Salvalaggio

**Affiliations:** 1 Academic Medicine Course, Universidade Positivo - Curitiba (PR), Brazil.; 2 Department of Medicine, Universidade Positivo - Curitiba (PR), Brazil.

**Keywords:** Transplantation, Organ donation, Public opinion, Tissue and organ procurement, Public health, Intensive care units

## Abstract

**Objective:**

To evaluate the knowledge and acceptance of the public and professionals working in intensive care units regarding organ donation after cardiac death.

**Methods:**

The three hospitals with the most brain death notifications in Curitiba were selected, and two groups of respondents were established for application of the same questionnaire: the general public (i.e., visitors of patients in intensive care units) and health professionals working in the same intensive care unit. The questionnaire contained questions concerning demographics, intention to donate organs and knowledge of current legislation regarding brain death and donation after cardiac death.

**Results:**

In total, 543 questionnaires were collected, including 442 from family members and 101 from health professionals. There was a predominance of women and Catholics in both groups. More females intended to donate. Health professionals performed better in the knowledge comparison. The intention to donate organs was significantly higher in the health professionals group (p = 0.01). There was no significant difference in the intention to donate in terms of education level or income. There was a greater acceptance of donation after uncontrolled cardiac death among Catholics than among evangelicals (p < 0.001).

**Conclusion:**

Most of the general population intended to donate, with greater intentions expressed by females. Education and income did not affect the decision. The type of transplant that used a donation after uncontrolled cardiac death was not well accepted in the study population, indicating the need for more clarification for its use in our setting.

## Abstract (pt)

**Objetivo:**

Avaliar o conhecimento e a aceitação da população e dos profissionais que trabalham em unidades de terapia intensiva sobre a doação de órgãos após morte cardíaca.

**Métodos:**

Foram elencados os três hospitais com mais notificações de morte encefálica em Curitiba e estabelecidos dois grupos de entrevistados pelo mesmo questionário: o público geral, ou seja, acompanhantes de pacientes em unidades de terapia intensiva, e profissionais de saúde que trabalhavam nas mesmas unidades de terapia intensiva. O questionário aplicado perguntou sobre dados demográficos, a intenção de doar órgãos e o conhecimento da legislação vigente, bem como sobre morte encefálica e doação após morte cardíaca.

**Resultados:**

No total, foram 543 questionários coletados, sendo 442 de familares e 101 de profissionais de saúde. Observou-se predomínio de mulheres e de católicos em ambos os grupos. O sexo feminino apresentou maior intenção de doar. Os profissionais de saúde tiveram um desempenho melhor na comparação de conhecimento. A intenção de doar órgãos foi significativamente maior no grupo de profissionais de saúde (p = 0,01). Não houve diferença significativa na intenção de doar com relação ao grau de instrução ou renda. Houve maior aceitação da doação após morte cardíaca não controlada entre os católicos, quando comparados com os evangélicos (p < 0,001).

**Conclusão:**

A maioria da população geral teve intenção de doar, sendo maior a intenção no sexo feminino. Escolaridade e renda não influenciaram em tal decisão. A modalidade de transplante que utiliza doação após morte cardíaca não controlada não teve boa aceitação na população estudada, apontando para a necessidade de mais esclarecimentos para o uso no nosso meio.

## INTRODUCTION

Solid organs for transplantation may be obtained from deceased donors with brain death or by donation after cardiac death (DCD).^(1-5)^ The criteria for defining cardiac death in DCD differ from those that define brain death, but potential donors also have irreversible damage and only become candidates for this type of donation when authorized by the family.^(4,6,7)^

Cardiac death can occur under different circumstances. The first DCD workshop held in Maastricht in 1995 identified four categories of DCD depending on the scenario in which there was irreversible respiratory or cardiac arrest. According to the Maastricht classification, types I (dead on arrival) and II (unsuccessful resuscitation) are categorized as "uncontrolled" DCD. Maastricht type III (awaiting cardiac death) and IV (cardiac arrest in a donor with brain death) have been referred to as "controlled DCD" because the patients are in the hospital. In 2000, type V was included, in which the critical patient's heart stops unexpectedly; this type was also categorized as uncontrolled DCD.^(2,7-9)^ Table 1 shows the different categories of this classification system.

**Table 1:** Maastricht Classification

Category I	Dead on arrival at hospital
Category II	Unsuccessful resuscitation
Category III	Anticipated cardiac arrest
Category IV	Cardiac arrest in donor with brain death
Category V	Unexpected arrest in critically ill patient

In category I, it is important that the exact time of death is recorded (documented by witnesses). This category is the most widely used type in uncontrolled donations. Category II requires that the patient be within the trauma service and that the resuscitation time be documented. In category III, the patient does not meet the criteria of brain death, which is in contrast to category IV, in which the patient is brain dead and suffers arrest.^(10-12)^

Prior to the current knowledge on brain death, DCD was the only method to obtain organs for transplantation. Currently, DCD is used in 27 countries in the European Union, United States, Canada, Australia, Japan, China, the Far East and some countries in South America.^(7,13,14)^ Some countries only use Maastricht type III and IV donations, and these donation types generally have little effect on the transplantation waiting list, whereas other countries use organs originating from Maastricht I donors, such as Spain. In Madrid, 234 patients were awaiting kidney transplantation in 1996. A new study performed in 2005 showed that this number had decreased to 32 patients.^(13,14)^ It should be taken into account that much of this success in Spain was due to the better training of teams in conducting the family interview. It is also important to note that approximately 33 people per million (pmp) of Spanish recipients still receive their organs from brain-dead donors and that only 3 pmp originate from asystole donation. Legislation in Brazil does not cover DCD. The harvesting of organs for donation may be performed only after brain death.^(8,15,16)^

In Brazil, the number of donations in 2014 increased by 7.6%, which was still approximately 5% lower than expected.^(8)^ Although initiatives such as staff training and a more effective system to increase the uptake of organ donation in brain death have not been exhausted, the use of DCD may be an alternative to help reduce transplant waiting lists. However, there are no reports concerning the opinions and knowledge of Brazilian society regarding DCD. Furthermore, it may be that neither the general population nor health professionals are aware of DCD procedures.

This study aimed to evaluate the knowledge and acceptance rate of the general population and health professionals working in intensive care units regarding organ donation after cardiac death.

## METHODS

The study was approved by the Research Ethics Committee of the *Universidade Positivo*; protocol no. 228286/2012). The questionnaire was applied between January and October of 2013.

According to data obtained from the Paraná Transplant Center, the top three hospitals accounting for the largest number of brain death notifications in the metropolitan region of Curitiba, Paraná (PR), were selected. The chosen hospitals were *Hospital do Trabalhador, Hospital Universitário Cajuru* and *Hospital Evangélico de Curitiba*. Over 70% of organ donors in the Curitiba metropolitan area are captured in these hospitals, which correspond to approximately 20% of the donors in the state of Paraná.

Two groups of respondents who answered the same questionnaire were established for the study. All surveyed respondents had a minimum age of 18 years and signed a free and informed consent form. Group A was composed of the general public (i.e., those visiting intensive care unit (ICU) patients in the three Curitiba hospitals) who could eventually encounter situations in which they might need to make a decision about organ donation. Group B (health professionals working in the ICUs) was composed of doctors, nurses, nursing technicians and assistants, physiotherapists, psychologists and pharmacists.

Group A was interviewed in the waiting rooms of the three hospitals' ICUs. The interviews were conducted during visiting hours, when the visitor was approached and invited to participate by answering a self-administered questionnaire. In Group B, health professionals who had worked in the ICU for at least six months were interviewed. This group was chosen because they were directly involved in organ harvesting situations. They were approached and invited to participate in the study in their work setting.

### Questionnaire

The self-administered questionnaire was developed by the researchers and divided into four parts: (1) demographic data collection (gender, age, profession, religion, family income and level of education); (2) intention or not to donate organs and knowledge of the law; (3) knowledge about brain death and cardiac death; and (4) acceptance of donation in hypothetical accident scenarios in which the respondent would have to decide about donation.

A controlled DCD scenario was used. Case A involved cardiopulmonary arrest occurring in a planned care withdrawal scenario (i.e., Maastricht type 3). Case B addressed an uncontrolled DCD scenario when cardiopulmonary arrest occurred unexpectedly (Maastricht types 1, 2 and 4). The scenarios were based on a method previously established by Volk et al.^(17)^

The questionnaire is presented in appendix 1.

### Statistical analysis

The sample size for the group of visitors was calculated by taking into account the number of ICU beds available at the locations where the study took place. In the health professionals group, all workers of the three ICUs were invited to participate in the study. Data were collected and the statistical analysis was performed with the aid of the Prism 5.0 package (GraphPad Prism, CA, USA). The Kolmorov-Smirnov test was used to verify data normality. Continuous variables were expressed as the mean ± standard deviation and compared using Student's *t* test. Categorical variables were expressed as percentages and compared using the Chi-square test or Fisher's exact test as appropriate. P values < 0.05 were considered statistically significant.

## RESULTS

Initially, a total of 715 questionnaires were distributed (600 to the general population and 115 to health professionals). In total, 543 questionnaires were completed correctly (442 in Group A and 101 in Group B). The total study participation acceptance rate was 75.9%; this rate was higher among the health professionals (87.8%) than the visitor population (73.6%). Most Group A respondents were children and spouses of patients hospitalized in the ICU. Questionnaires returned blank or incomplete were excluded from the study.

The respondent demographics are shown in table 2. The mean age of Group A was 35.7 ± 13.10 years and of Group B was 35.6 ± 9.33 years (p = not significant).

**Table 2:** Respondent demographics

General public	N (%)	Health professionals	N (%)
Sex			
Male	184 (42)		27 (27)
Female	258 (58)		74 (73)
Profession			
Homemaker	64 (14.4)	Nursing assistant or technician	53 (52)
Self-employed	99 (22.4)	Nurse	24 (24)
Student	23 (5)	Doctor	18 (18)
Unemployed	12 (2.7)	Other	6 (6)
Retired	12 (2.7)	---	---
Other	232 (52)	---	---
Religion			
Catholic	247 (56)		60 (59)
Evangelical	154 (34.9)		25 (25)
Other	40 (9)		16 (16)
Income (MW)			
Up to 1	55 (12)		4 (4)
1 - 3	197 (45)		28 (28)
3 - 5	110 (25)		29 (29)
Above 5	80 (18)		40 (39)
Education level			
Primary	125 (28.3)		7 (7)
Secondary	197 (44.4)		32 (31)
Higher	120 (27)		62 (62)

MW - minimum wage.

Both groups contained more women than men (Group A = 58%; Group B = 73%). The predominant religion was Catholic (Group A = 56%; Group B = 59%). With respect to income, 45% of the participants in Group A had an average income of one to three times the minimum monthly wage (between US$290.00 and 870.00), whereas in Group B, 39% of the participants had an average monthly income of more than five times the minimum monthly wage range (> US$1,450.00). There were more respondents with higher education levels in Group B (62%), whereas in Group A, the secondary education level predominated, with 44%.

The data on general knowledge regarding organ donation are shown in table 3. As expected, the health professionals scored higher. In Group A, 60% of the participants reported that they did not know the current legislation on donations in Brazil, whereas in Group B, 31% did not know. The majority in both groups answered correctly when asked who authorized organ donations (Group A = 84%; Group B = 96%). Regarding knowledge on the concept of brain death, 50% of the participants in Group A and 65% in Group B answered correctly.

**Table 3:** General knowledge about organ donation in the studied groups

	General public N (%)	Health professionals N (%)	p value
Would donate all organs	351 (80)	74 (73.2)	0.17
Law 9434/1997			
Knows in detail	27 (6)	16 (16)	
Knows but not in detail	147 (33)	54 (53)	
Does not know	268 (60)	31 (31)	0.001
Donor availability			
More than sufficient	8 (1.8)	0 (0)	
Sufficient	4 (0.9)	3 (3)	
Insufficient	320 (72)	86 (85)	
Does not know	110 (24.8)	12 (12)	0.0047
Who authorizes organ removal			
Correct answer	373 (84)	97 (96)	0.002

Table 4 shows the intention to donate and the acceptance of DCD. The intention to donate organs was significantly higher in the health professionals group (Group A = 58%; Group B = 74%, p = 0.01). An interesting study finding was that all of the doctors interviewed intended to donate. There was no significant difference in the intention to donate in terms of education level or income. With regard to religion, there was greater acceptance of DCD among Catholics than among evangelicals. More females intended to donate (60% versus 51%; p = 0.07).

**Table 4:** Intention to donate and acceptance of donation after cardiac death in the studied groups

	Intention to donate N (%)	Believes knows the concept of cardiac arrest N (%)	Would accept uncontrolled DCD* N (%)	Would accept controlled DCD† N (%)
General public (N = 442)	260 (58)	242 (54)	267 (60)	336 (76)
Religion				
Catholic (N = 247)	143 (57)	145 (58)	168 (68)	192 (77)
Evangelical (N = 154)	93 (60)	76 (49)	72 (46)	105 (68)
p value	0.62	0.06	0.001*	0.033*
Educational level				
Primary (N = 125)	72 (57)	58 (46)	75 (60)	93 (74)
Secondary (N = 197)	114 (57)	114 (57)	124 (62)	145 (73)
Higher (N = 120)	74 (61)	70 (58)	68 (56)	98 (81)
p value	0.55	0.82	0.43	0.19
Sex				
Male (N = 184)	95 (51)	103 (55)	119 (64)	141 (66)
Female (N = 258)	155 (60)	138 (53)	148 (57)	195 (74)
p value	0.07	0.68	0.12	0.79
Health professionals (N = 101)	75 (74)	96 (95)	50 (49)	69 (68)
Doctors (N = 18)	18 (100)	17 (94)	10 (55)	15 (83)
Other health professionals (N = 83)	57 (68)	79 (95)	40 (48)	53 (63)
p value	0.01	0.86	0.54	0.19

DCD - donation after cardiac death.

* uncontrolled DCD - cardiopulmonary arrest occurs unexpectedly;

† controlled DCD - cardiopulmonary arrest occurs in a scenario of planned withdrawal of care.

* Chi-square test.

## DISCUSSION

This study represents a pioneering approach in Brazil to view acceptance of a transplant modality that is little known in the country (DCD). The national literature on the subject is scarce, and one of the reasons the study was performed was to introduce the debate on DCD use in Brazil. This type of organ donation has evolved in different ways in different countries.^(15,16)^ DCD has increased steadily in the United States and is now responsible for approximately 10% of donations.^(18)^ In Japan, DCD remains the main source of organs for transplantation from deceased donors.^(14)^ In Europe, DCD is increasingly accepted and used but is still limited to a few countries.^(13,19)^

One finding that drew attention in this study was that 60% of the general population reported that they did not know the current legislation on organ donation in Brazil. In the health professionals group, 31% reported not knowing. Conversely, the majority of both groups were correct when asked who authorized an organ donation. Additionally, when asked what they knew about brain death, only 50% of the general population reported that they were knowledgeable. This result was different from that found by Coelho et al.,^(20)^ who reported 86.7% correct answers. Among health professionals, 65% understood the concept of brain death, which could be considered a low number and reinforced the need for clarification of the public and health professionals regarding organ transplants in general. The heterogeneity of education levels among professionals in the study should be noted, as seen from the demographics.

When the intention to donate was compared between the general population group and the health professionals, a significant increase was observed in the intention to donate among health professionals, and all doctors who answered the questionnaire intended to donate. This finding may be related to the daily lives of health professionals working in the ICU, who in addition to experiencing the drama of patients and families who are waiting for an organ transplant have more specific knowledge on the subject and may possibly be more open to this practice. Regarding the intention to donate, the percentage in Group A (58%) was lower than the percentage reported in another study conducted in Curitiba, in which 87.8% of respondents reported being donors.^(20)^ In a study conducted in the state of Pará, 84.6% of respondents (general population) were in favor of donation. In that study, 85.3% of respondents believed that the doctor could be mistaken in the diagnosis of brain death.^(21)^ Notably, these Brazilian studies investigated the intention to donate after brain death, and there was an absence of studies related to DCD.

In the present study, a predominance of females was observed in both groups, which was also the case in a study conducted in Curitiba.^(20)^ In relation to Group B, almost all of the professionals working in the ICUs of the hospitals studied were interviewed, and 76% of this group consisted of nursing professionals with a clear female predominance. Regarding the intention to donate, there was a greater number of positive responses among females (61% versus 50% in men, p = 0.07). This predominance can be partially explained by the fact that most caregivers of patients being treated in the ICU are female.^(22)^ It was also evident that the education levels and family incomes did not significantly affect the intention to donate in the study groups. These parameters also did not affect responses in the study conducted by Teixeira et al. in northern Brazil.^(21)^

When evaluating the hypothetical DCD situation, we found that acceptance of controlled DCD was significantly higher than acceptance of uncontrolled DCD for both groups. This result is understandable because uncontrolled DCD represents part of a sudden and unexpected situation for the family.^(15,16)^ In a study conducted in the United States using the same questions, acceptance of controlled DCD was slightly higher than acceptance of uncontrolled DCD and was also higher than acceptance in cases of brain death (70%, 69% and 66%, respectively).^(18)^ Another interesting finding was the greater acceptance in Group A of both types of DCD. The greater complexity of the clinical case presented in the questionnaire in relation to the other questions may have caused difficulties in understanding and interpretation in Group A given that this group has less education and specific knowledge than Group B. Among the health professionals, there was greater acceptance by doctors than by other professionals (55% versus 48% for uncontrolled DCD and 83% versus 63% for controlled DCD). Perhaps being better informed on the subject positively affects the result. In a similar study conducted in the United States, only 46% of health professionals and hospital-based managers accepted organ donation via uncontrolled DCD.^(19)^

When compared by religion, although no difference was observed in the intention to donate, a greater acceptance of donation was noted among Catholics than among evangelicals, both for cases of uncontrolled and controlled DCD. This finding is curious because neither religion declares any restrictions related to transplants. Coelho et al.^(20)^ also found no differences between religions in the intention to donate. However, in a recent study in England, the absence of religion, Anglican Christianity, Buddhism and Hinduism were positively associated with a desire to donate all organs.^(23)^ The use of uncontrolled DCD gives rise to a number of procedural, medical, economic, legal and ethical challenges. However, according to a recent meta-analysis, uncontrolled DCD is a viable option for increasing organ donation because there are reports of good results for kidney, liver and lung transplants.^(24)^

In Brazil, there are still legal, ethical and technical obstacles to this type of donation. There is a shortage of organs and a need to associate transplantation practices with advances in intensive care. To improve this scenario, it is essential that the country's current donation system be improved, with greater notification of brain death, a reduction in the number of cases in which there is "organ waste" linked to family refusal and cardiac arrest rates (e.g., when DCD could be an option for donation) and the resolution of logistical problems.^(13)^ In addition to these immediate actions, which are necessary to expand the donor pool, in the future, alternative modes could be included, such as asystolic patient donation. Therefore, we believe that this evaluation of general knowledge about organ donation and the level of acceptance of situations involving DCD scenarios in our population can contribute to an initial discussion about this form of transplant.

Actions that contribute to an effective increase in the notification of potential donors, viability and the use of organs and tissues are always necessary to try and minimize mortality on the waiting list.^(8)^ Information is the main factor in organ donation signup, and educational measures should be instituted. In this study, we noted that most of the population expressed the intention to donate organs, but there was still little knowledge regarding legislation.

DCD is a topic that is still relatively unknown in our setting. Studies in other countries have shown that the prospect of enlarging the donor pool with asystole is at most 3 pmp in 10 years as opposed to 10 to 15 pmp if the hard work of reducing the number of families that refuse to donate, cardiac arrests and incorrectly attributed contraindications is performed. Furthermore, these measures have an extremely low financial cost compared to the greater complexity of procedures involved in DCD. There is much to be done to reduce transplant waiting lists in Brazil, and it is appropriate that the use of DCD is discussed in all settings involved in organ donation. The competent authorities must evaluate the need to use DCD to increase organ harvesting and if applicable institute legislation regarding this type of donation.

## CONCLUSION

The majority of the population studied intended to donate, and more females intended to donate than males. Education, religion and income did not affect this decision. The type of transplant that used a donation after uncontrolled cardiac death was not well accepted in the study population.
